# Forecast and analysis of aircraft passenger satisfaction based on RF-RFE-LR model

**DOI:** 10.1038/s41598-022-14566-3

**Published:** 2022-07-01

**Authors:** Xuchu Jiang, Ying Zhang, Ying Li, Biao Zhang

**Affiliations:** 1 School of Statistics and Mathematics, Zhongnan University of Economics and Law, Wuhan, 430073 China; 2 Department of Scientific Research, Zhongnan University of Economics and Law, Wuhan, 430073 China; 3 School of Computer Science, Liaocheng University, Liaocheng, 252059 China

**Keywords:** Applied mathematics, Computer science, Aerospace engineering

## Abstract

Airplanes have always been one of the first choices for people to travel because of their convenience and safety. However, due to the outbreak of the new coronavirus epidemic in 2020, the civil aviation industry of various countries in the world has encountered severe challenges. Predicting aircraft passenger satisfaction and excavating the main influencing factors can help airlines improve their services and gain advantages in difficult situations and competition. This paper proposes a RF-RFE-Logistic feature selection model to extract the influencing factors of passenger satisfaction. First, preliminary feature selection is performed using recursive feature elimination based on random forest (RF-RFE). Second, based on different classification models, KNN, logistic regression, random forest, Gaussian Naive Bayes, and BP neural network, the classification performance of the models before and after feature selection is compared, and the prediction model with the best classification performance is selected. Finally, based on the RF-RFE feature selection, combined with the logistic model, the factors affecting customer satisfaction are further extracted. The experimental results show that the RF-RFE model selects a feature subset containing 17 variables. In the classification prediction model, the random forest after RF-RFE feature selection shows the best classification performance. Finally, combined with the four important variables extracted by RF-RFE and logistic regression, further discussion is carried out, and suggestions are given for airlines to improve passenger satisfaction.

## Introduction

With the continuous improvement of people's living standards, civil aviation industry customer groups are growing, and people have put forward higher requirements for aviation service quality. In addition, the COVID-19 outbreak has hit most industries around the world, bringing many to a standstill. Movement restrictions and travel bans have had a serious impact on the transport sector, especially the airline sector, and the quality of airline service has become a more important factor for people's choice^1^. In a fierce competitive environment, the aviation industry should also develop from a simple transport role to service. Improving service quality is an important part of competitiveness and an important guarantee for sustainable and healthy development^2,3^. Therefore, airlines should investigate passengers' satisfaction with various services and overall satisfaction in a timely manner and accurately understand the service quality of existing services. In addition, airlines should accurately grasp the main factors affecting passenger satisfaction, and formulate corresponding strategies to improve service quality, to maximize the overall passenger satisfaction with the airline and improve passenger loyalty.

Based on the above challenges, this study uses the full-service passenger information and satisfaction survey results as the research object. While using machine learning algorithms to predict passenger satisfaction, the main factors affecting passenger satisfaction are further studied, and the priority of the factors are given.

This study provides a reference for airlines to accurately predict the overall satisfaction of passengers with the company's services and understand the main factors by using passenger personal and service satisfaction survey information. This study serves as a reference for airlines to use customer evaluation-driven service evaluation methods, and provides support for airlines to improve their competitiveness.

### Airline passenger satisfaction

Customer satisfaction is increasingly recognized as a determinant of business performance and a strategic tool for gaining competitive advantage. Cardozo first put forward the viewpoint of customer satisfaction in 1965 and introduced it into the field of marketing for the first time^4^, and the theory of customer satisfaction has also made continuous development. High and stable customer satisfaction is considered an important determinant of an organization's long-term profitability. Yeung^5^ found a positive relationship between customer satisfaction and a range of financial performance indicators by using the American Consumer Satisfaction Index. Research has also shown a significant moderate-to-strong association between satisfaction and a company's financial and market performance. More specifically, customer satisfaction is strongly linked to retention, revenue, earnings per share, and stock price^6^.

For the aviation industry, some studies have used aviation services to build an index model for passenger satisfaction. Based on the combination of China's customer satisfaction index model and the actual situation of China Southern Airlines' satisfaction management, Zhang^7^ designed the China Southern Airlines customer satisfaction evaluation index and proposed nine secondary indicators with air transport characteristics: flight operation quality satisfaction degree, ticketing service satisfaction, ground service satisfaction, air service satisfaction, arrival station service satisfaction, irregular flight service satisfaction, consumption value perception, overall satisfaction, and customer loyalty.

There are also studies using flight data or text reviews to predict passenger satisfaction. Sankaranarayanan et al.^8^ used a logistic model tree (LMT) machine learning approach to predict passenger satisfaction levels based on factors such as airport punctuality, number of flights, punctuality rankings, average delays, and queue times for inferring passenger perceptions of punctuality and delay-related event satisfaction. Kumar et al.^9^ predicted passengers' positive or negative attitudes by textually evaluating passengers' flight reviews.

Many studies have discussed the impact of passenger satisfaction on modern businesses, and how passenger satisfaction affects business performance and development in the airline industry, so it is crucial to obtain timely and accurate information on passenger satisfaction with airlines.

#### Service quality and passenger satisfaction

To achieve high levels of customer satisfaction, service providers should provide high levels of service quality, as service quality is often considered a prerequisite for customer satisfaction^10^^.^ Since passengers are the direct recipients of services, service quality indirectly affects enterprise development by affecting passenger satisfaction. Therefore, airlines can understand the quality of the services provided by passengers' satisfaction with each service, check the services, and then improve the service quality. Park et al.^11^ show that airlines that provide services that meet customer expectations enjoy higher levels of passenger satisfaction and value perception. Jiang et al.^12^ took China Eastern Airlines (CEA) as a case study to investigate the domestic passengers of China Eastern Airlines at Wuhan Tianhe International Airport, China. Hu et al. ^13^found that poor service quality can lead to customer dissatisfaction by using the Kano model to design a quality risk assessment model. Chow et al.^14^ studied the relationship between customer satisfaction measured by customer complaints and service quality using fixed-effect Tobit analysis, discussed the seasonality of customer satisfaction, and compared customer satisfaction between state-controlled and nonstate-controlled operators.

In many studies, it is proposed to combine passenger satisfaction information to construct a service quality evaluation framework^15^. Therefore, the influence mechanism between service quality and satisfaction should be that service quality affects satisfaction, and satisfaction is a direct and effective indicator of service quality. Therefore, this study combines service quality with passenger satisfaction, predicts overall satisfaction with passengers' ratings of service quality and uses satisfaction information to analyze airline service quality.

#### Important service attributes

For the aviation industry, it is more efficient to improve customer satisfaction by accurately understanding the main factors affecting passenger satisfaction and making improvements based on service priorities. Several studies have investigated the main factors influencing passenger satisfaction. By constructing a nested logit model of airport-airline choice in the "two-step" decision-making process of air passengers, Suzuki^16^ determined that the factors that play an important role in airline choice are ticket price, frequency of flight service provided to desired destinations and frequent flyer membership. Tsafarakis et al.^17^ proposed that the improvement of onboard entertainment onboard Wi-Fi services can improve airline passenger satisfaction according to the multi-standard satisfaction analysis method. Hess^18^ looked at these factors separately for several market segments and concluded that visit times, flight times and airfares are important for both business and holidaymakers. With the development of text mining technology, some researchers have mined customer comment text on web pages to analyze passenger satisfaction and the main influencing factors. Lucini et al.^19^ used a text mining method to analyze online customer comments and predict passengers' attitudes and concluded that first-class passengers should be provided with customer service for different reasons. Provide comfort for premium economy passengers; Conclusions on checked baggage and wait times for economy passengers Cabin staff, in-flight service and cost performance were found to be the three most important dimensions in the prediction of airlines recommended by passengers. According to an online review study by Brochado^20^, on-board service, airport operation, ground service and other factors have a significant impact on service quality assessment by using Leximancer to perform quantitative content analysis of airline passenger web reviews.

Many previous studies evaluated passenger satisfaction by statistical tests, building a satisfaction index system^7^ or text analysis^20^. Then, this study uses machine learning to determine the main factors affecting satisfaction based on the data of airline passenger satisfaction surveys and gives priority to the services that airlines need to pay attention to, providing strategic support for companies committed to improving passenger satisfaction.

### Feature selection based on RF-RFE

Feature selection refers to removing redundant or irrelevant features from a set of features. Whether the samples contain irrelevant or redundant information directly affects the performance of the classifier, so it is very important to choose an effective feature selection method. Guyon^21^ reviewed the existing variable feature selection methods: filtering, embedding and packaging. The filtering method sorts the features in the preprocessing step and is independent of the learning algorithm, which means that the selected features can be transferred to any modeling algorithm. The filter can be further classified according to the filtering measures used, i.e., information, distance, dependence, consistency, similarity and statistical measures. The embedded method takes feature selection as part of the implementation of the modeling algorithm. The features of method selection depend on the learning algorithm. Two typical examples are lasso and various decision tree-based algorithms. Wrapper uses an independent algorithm to train the prediction model for candidate feature subsets and uses greedy strategies such as forward or backward to identify the optimal feature subset from all possible subsets in the learning process.

Recursive feature elimination (RFE) is a sequence backward selection algorithm belonging to the wrapper. This method uses a specific underlying algorithm to continuously reduce the scale of the feature set through recursion to select the required features. Guyon^21^ proposed RFE based on the SVM model and proved that SVM-RFE achieved very good results in the process of gene selection, which has become a widely used method in gene selection research^22^. Marcelo MCA combined RFE with logistic regression (LR) and a support vector machine (SVM) to select the features of tobacco spectral information, which improved the prediction accuracy of the model^23^. Wei^24^ and others proposed the SVM-RFE-SF method, which divides the original gene set into several gene subsets, and RFE divides the genome with the smallest score of the sorting criteria, which effectively solves the problem of heavy calculation caused by eliminating only one gene at a time.

The feature selection method using random forests in most studies is the wrapper^25^. In the research of feature selection based on RF-RFE, Wu combined RF with RFE and used the importance ranking output of the RF algorithm to select variables^26^. Chen tested two completely different molecular biology data sets and found that using RF-RFE feature selection improves the quality of the model and makes the model construction process more efficient than the model without any feature selection^27^. Shang et al.^28^ used the RF-RFE algorithm to select important variables from the initial variables for evaluating traffic event detection and took important variables as input to prove that the model has better performance.

This study uses machine learning models to predict the overall satisfaction of passengers with the full service provided by the airline. Combined with feature selection, the prediction models before and after feature extraction are compared, and the best prediction model is selected. We aim to select the important factors that affect passenger satisfaction. The preliminary feature selection is combined with the logistic model for further selection, and the model results are used to prioritize the influencing factors.

## Methodology

In this section, we present a detailed introduction to the preliminary feature selection method RF-RFE (random forest-based recursive feature elimination) and various classification models used for passenger satisfaction in this study.

### Recursive feature elimination based on random forest

#### RF

The RF proposed by Breiman^29^ is a parallel integration algorithm based on a decision tree. Because of its relatively good precision, robustness and ease of use, it has become one of the most popular machine learning methods. The decision tree may be completely different due to the small change in data, so it is not stable enough. The RF reduces the variance brought by a single decision tree, improves the prediction performance of the general decision tree, and can give the importance measurement of variables, which brings substantial improvement to the decision tree model.

RF uses a decision tree as the base learner to construct a bagging ensemble. Bagging is a parallel integrated learning algorithm based on a self-help sampling method. Each sampling set is used to train a base learner, and then these base learners are combined. When combining the prediction output, the simple voting method is usually used for the classification task.

Let the training set $D=\left\{\left({x}_{1},{y}_{1}\right),\left({x}_{2},{y}_{2}\right),\dots ,\left({x}_{n},{y}_{n}\right)\right\}$, and the prediction result of the new sample is Eq. (1):

1 $$f\left( x \right) = \mathop {{\text{argmax}}}\limits_{{y \in {\mathcal{Y}}}} \mathop \sum \limits_{{t = 1}}^{T} {\mathbb{I}}\left( {h_{t} \left( x \right) = y} \right)$$

where *y* is the output category set, ${h}_{t}(x)$ is the prediction result of the new sample *x* by the *t*-th learner, and *y* is the real category of the sample.

RF introduces the selection of random attributes on the basis of bagging integration. Different from selecting an optimal attribute when a single decision tree divides attributes, RF adopts the method of random selection for each node attribute set in the decision tree, first randomly selects an attribute subset from all attributes, and then selects an optimal attribute from the subset. Therefore, based on the sample disturbance brought by bagging, the RF further introduces attribute disturbance, which increases the generalization performance of the integration. The algorithm description of RF is shown in Table 1.

**Table 1:**
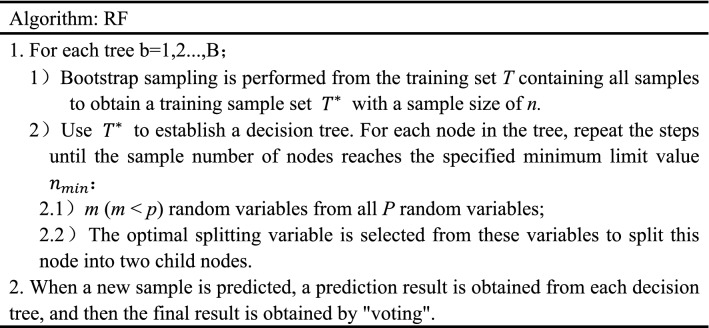
RF algorithm.

#### Importance of RF characteristics

The importance measurement indicators based on RF include the mean decrease impurity (MDI) based on the Gini index and the mean decrease accuracy (MDA) based on OOB data^30^. This method uses the frequency of attributes in the RF decision tree to reflect the importance of features. This paper chooses the MDI method based on the Gini index to measure the importance of features.

When constructing the CART decision tree, RF takes the attribute with the largest Gini gain as the splitting attribute by calculating the Gini gain of all attributes of the node. Gini represents the probability that a randomly selected sample in the sample set is misclassified, let ${p}_{k}$ be the proportion of class *k* samples, and the calculation equation is Eq. (2):

2 $$ Gini\left( p \right) = \mathop \sum \limits_{{k = 1}}^{K} p_{k} \left( {1 - p_{k} } \right) = 1 - \mathop \sum \limits_{{k = 1}}^{K} p_{k}^{2} $$

The Gini gain obtained by dividing the data set according to attribute *a* is Eq. (3):

3 $$ Gini\left( {D,a} \right) = Gini\left( D \right) - \mathop \sum \limits_{v}^{V} \frac{{\left| {D^{v} } \right|}}{{\left| D \right|}}Gini\left( {D^{v} } \right) $$

where V is the number of value categories of attribute *a* and $\left|{D}^{v}\right|$ is the number of value categories of attribute *a*.

Based on the calculation of feature importance, the specific steps are as follows:

1. For each decision tree, the node where feature $\propto$ appears is set A, and the change in the Gini index before and after node *i* branch is calculated as follows Eq. (4):
  4 $$\Delta Gini = Gini\left( i \right) - Gini\left( l \right) - Gini\left( k \right)  $$
  where $Gini\left(l\right)$ and $Gini(k)$ are the Gini index of the new node after branching.
2. The importance of feature $\propto$ in the tree is shown in Eq. (5):
  5 $$ IM_{ \propto }  = \mathop \sum \limits_{{a \in A}} \Delta Gini  $$
  where *a* is the node where feature $\propto$ appears.
3. Suppose n is the number of decision trees, and the importance of feature $\propto$ is Eq. (6):
  6 $$ IMPORTANCE\left(  \propto  \right) = \mathop \sum \limits_{N} IM_{ \propto }$$
  Then, normalize the importance of all features in Eq. (7):
  7 $$IM\left(  \propto  \right) = \frac{{IMPORTANCE\left(  \propto  \right)}}{{\mathop \sum \nolimits_{i}^{c} IMPORTANCE\left( i \right)}} $$
  where *c* is the number of features.
4. The larger the $IM\left(\propto \right)$ value is, the more important the feature is to the result prediction, that is, the higher the importance of the feature.

#### Recursive feature elimination based on RF

**Figure 1:**
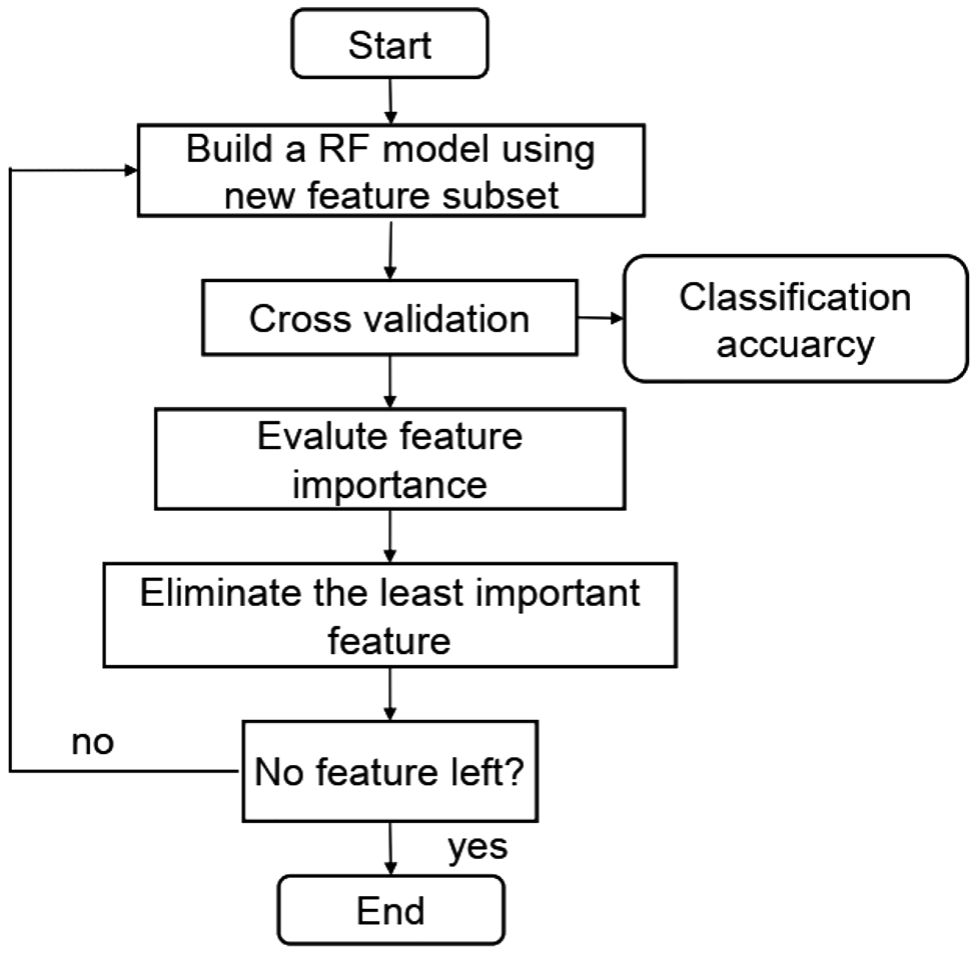
The RF-RFE flow.

RF-RFE uses RF as an external learning algorithm for feature selection, calculates the importance of features in each round of feature subset, and removes the features corresponding to the lowest feature importance to recursively reduce the scale of the feature set, and the feature importance is constantly updated in each round of model training. Based on the selected feature set, this study uses cross validation to determine the feature set with the highest average score based on classification accuracy. The algorithm flow chart is shown in Fig. 1.

The RF-RFE flow is as follows:

1. Bootstrap sampling is carried out from the training set *T* containing all samples to obtain a training sample set ${T}^{*}$ with a sample size of *n*. The decision tree is established by using ${T}^{*}$, and a total of *b* decision trees are generated by repeating this process;
2. The prediction results of each decision tree are combined by "voting", and the effect of the RF regression model is evaluated based on classification accuracy by using the fivefold cross validation method;
3. Calculate and sort the importance $IM\left(\propto \right)$ of each feature $\propto$ in the feature set based on MDI;
4. According to the backward selection of the sequence, delete the feature with the lowest feature importance, and repeat steps 1–3 for the remaining feature subset until the feature subset is empty. According to the cross-validation results of each feature subset, the feature subset with the highest classification accuracy is determined.

### Satisfaction prediction based on machine learning algorithm

According to whether the processed data are marked artificially, machine learning can be generally divided into supervised learning and unsupervised learning. Supervised learning data sets include initial training data and manually labeled objects. The machine learning algorithm learns from labeled training data sets, tries to find the pattern of object division, and takes labeled data as the final learning goal. Generally, the learning effect is good, but the acquisition cost of labeled data is high. Unsupervised learning processes unclassified and unlabeled sample set data without prior training, hoping to find the internal rules between the data through learning to obtain the structural characteristics of the sample data, but the learning efficiency is often low. The satisfaction status in this study is the data set label. In the training process, the supervised machine learning algorithm learns the corresponding relationship between features and labels and applies this relationship to the test set for prediction.

#### k-nearest neighbors (KNN)

**Figure 2:**
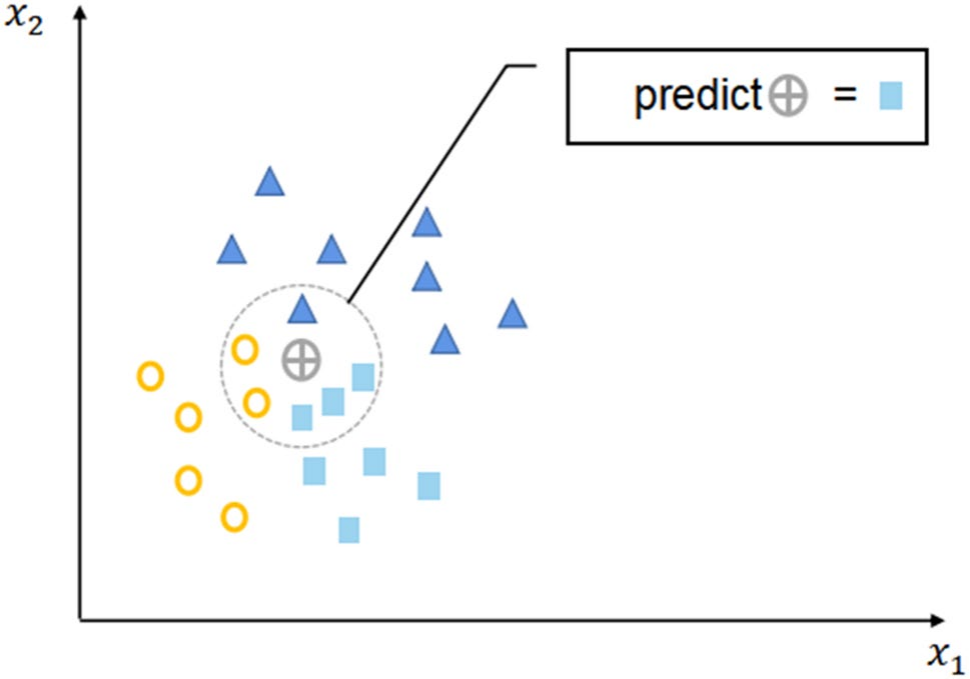
KNN.

KNN is a supervised learning algorithm. Because the training time overhead is zero, it is also representative of "lazy learning"^31^. K-nearest neighbor has been used as a nonparametric technique in statistical estimation and pattern recognition. The working principle is as follows: for a given new sample, find the K samples closest to the sample in the training set based on a certain distance measurement and take the number of categories with the largest number of K samples as the category of the new sample. The samples are not processed in the training stage, so it belongs to "lazy learning". As shown in Fig. 2, if there are 3 squares, 2 circles and 1 triangle around a data point, it is considered that the data point may be square. The parameter K in KNN is the number of nearest neighbors in majority voting.

#### LR

LR is used to evaluate the relationship between dependent variables and one or more independent variables, and the classification probability is obtained by using logical functions^32^. It is a learning algorithm with a logistic function as the core. A logistic function is used to compress the output of the linear equation to (0, 1). The logistic function is defined as Eq. (8):

8 $$Logistic\left( z \right) = ~\frac{1}{{1 + e^{{ - z}} }}$$

Consider the binary classification problem, given the data set $D=\left({x}_{1},{y}_{1}\right),\left({x}_{2},{y}_{2}\right),\dots ,\left({x}_{N},{y}_{N}\right),{x}_{I}\subseteq {R}^{n},{y}_{i}\in \mathrm{0,1},i=\mathrm{1,2},\cdots ,N$*.*

*P* is the probability that the sample is a positive example, and the coefficient in the following formula is determined by LR through the maximum likelihood method ${\beta }_{0},{\beta }_{1},\cdots ,{\beta }_{k}$ to make an estimate [Eqs. (9) and (10)]:

9 $$logit\left( p \right) = ~log\left( {\frac{p}{{1 - p}}} \right) = \beta _{0}  + \beta _{1} x_{1}  +  \cdots  + \beta _{k} x_{k}$$

10 $$p = \frac{{\exp \left( {\beta _{0}  + \beta _{1} x_{1}  +  \cdots  + \beta _{k} x_{k} } \right)}}{{1 + \exp \left( {\beta _{0}  + \beta _{1} x_{1}  +  \cdots  + \beta _{k} x_{k} } \right)}}$$

When *P* is greater than the preset threshold, the sample is divided into positive examples, and vice versa.

$\frac{p}{1-p}$ is called the odds ratio (odds), which refers to the ratio of the probability of event occurrence to the probability of event nonoccurrence. The logarithm of the winning rate is linear with the coefficient of the variable. When the features have been standardized, the greater the absolute value of the coefficient, the more important the feature is. If the coefficient is positive, this characteristic is positively correlated with the probability that the target value is 1; if the coefficient is negative, this characteristic is positively correlated with the probability that the target value is 0.

#### Gaussian Naive Bayes (GNB)

Naive Bayes (NB) is a direct supervised machine learning algorithm^33^. The NB classifier is based on the Bayesian probability theorem and predicts future opportunities according to previous experience. NB assumes that the input variables are conditionally independent [Eq. (11)].

11 $$ P\left( {Y = y_{k} {\text{|}}X_{1} , \ldots ,X_{n} } \right) = \frac{{P\left( {Y = y_{k} } \right)P\left( {X_{1} , \ldots ,X_{n} {\text{|}}Y = y_{k} } \right)}}{{\mathop \sum \nolimits_{j} P(Y = y_{j} )P\left( {X_{1} , \ldots ,X_{n} {\text{|}}Y = y_{k} } \right)}} = \frac{{P\left( {Y = y_{k} } \right)\mathop \prod \nolimits_{i} P\left( {X_{i} {\text{|}}Y = y_{k} } \right)}}{{\mathop \sum \nolimits_{j} P(Y = y_{j} )\mathop \prod \nolimits_{j} P\left( {X_{i} {\text{|}}Y = y_{j} } \right)}}$$

where *X* is the input vector $({X}_{1},{X}_{2},\dots ,{X}_{n})$ and *Y* is the output category.

On the basis of NB, GNB further assumes that the prior probability of the feature is a Gaussian distribution, that is, the probability density function is as follows in Eq. (12):

12 $$ P\left( {x_{i}  = x{\text{|}}Y = y_{k} } \right) = \frac{1}{{\sqrt {2\pi \delta _{{ik}}^{2} } }}e^{{ - \frac{1}{2}\left( {\frac{{x - \mu _{{ik}} }}{{\delta _{{ik}} }}} \right)^{2} }}  $$

For a given test set sample $\mathrm{X}=({\mathrm{X}}_{1},{\mathrm{X}}_{2},\dots ,{\mathrm{X}}_{\mathrm{n}})$, calculate *P* [Eq. (13)]:

13 $$ P\left( {Y = y_{k} } \right)\mathop \prod \limits_{i} P\left( {X_{i} |Y = y_{k} } \right),\quad k = 1,2, \ldots ,K $$

To determine the class of the sample *y* [Eq. (14)]:

14 $$ y = \mathop {argmax}\limits_{{y_{k} }} P\left( {Y = y_{k} } \right)\mathop \prod \limits_{i} P\left( {X_{i} {\text{|}}Y = y_{k} } \right) $$

#### RF

**Figure 3:**
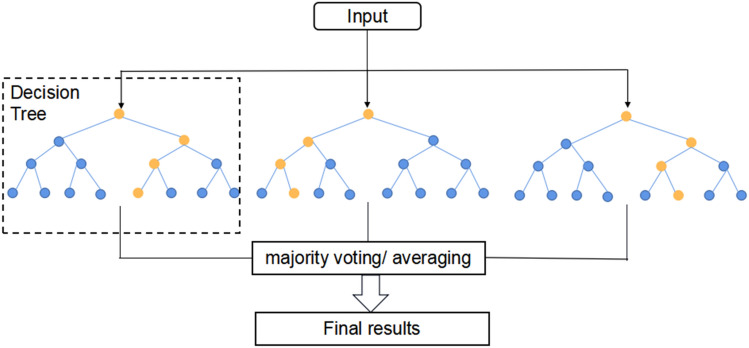
Working principle of RF.

The working principle of RF^34^ is to combine the results of each decision tree, as shown in Fig. 3. This strategy has better estimation performance than a single random tree: the estimation of each decision tree has low deviation but high variance, but clustering realizes the trade-off between overall deviation and variance and provides the importance of prediction variables to the prediction of result variables. RF has good prediction performance in practical applications and can be used to address multiclass classification problems, category variables and sample imbalance problems.

#### Backpropagation neural network (BPNN)

BPNN is one of the most widely used neural network models and is a typical error backpropagation algorithm^35^. Since the emergence of BPNNs, much research has been done on the selection of activation functions, the design of structural parameters and the improvement of network defects. The main idea of the BP algorithm is to divide the learning process into two stages: forward transmission and reverse feedback. In the forward transmission stage, the input sample reaches the output layer from the input layer through the hidden layer, and the output end forms an output signal. In the backpropagation stage, the error signals that do not meet the precision requirements are spread forward step by step, and the weight matrix between neurons is corrected through the pre-adjustment and post-adjustment cycles. When the iteration termination condition is met, the learning stops.

1. Forward transmission

First, the input vector of the sample is *X*, *T* is the corresponding output vector, *m* is the number of neural units in the input layer, and *P* is the number of nodes in the output layer:

$$ \begin{aligned}   X &  = \left( {x_{1} , \ldots ,x_{m} } \right) \\    T &  = \left( {T_{1} , \ldots ,T_{p} } \right) \\  \end{aligned}  $$

The calculation process equation of the forward transmission output layer is Eq. (15):

15 $$ I_{j}  = \mathop \sum \limits_{{i = 1}}^{m} w_{{ij}} x_{i}  + \theta _{j}  $$

where *j* represents the node of the hidden layer, *w* is the weight matrix between the input layer node and the hidden layer node, ${\theta }_{j}$ is the threshold of node *j*, and the output value of node *j* is Eq. (16):

16 $$ O_{j}  = f\left( {I_{j} } \right) $$

where *f* is called the activation function, which is the processing of the input vector. The function can be linear or nonlinear.

(2) Reverse feedback

Calculate the error between the true value of the sample and the output value of the sample. For the problem of second classification, two neural units are often used as the output layer. If the output value of the first neural unit of the output layer is greater than that of the second neural unit, it is considered that the sample belongs to the first category (Eq. (17)):

17 $$ E_{i}  = O_{i} \left( {1 - O_{i} } \right)\left( {T_{i}  - O_{i} } \right) $$

The error of the middle hidden layer is accumulated by weight through the node error of the next layer (Eq. (18)):

18 $$ E_{j}  = O_{i} \left( {1 - O_{i} } \right)\mathop \sum \limits_{k} E_{k} W_{{jk}}  $$

where ${E}_{k}$ is the error of the *k*-th node of the next layer and ${W}_{jk}$ is the weight from the *j*-th node of the current layer to the *k*-th node of the next layer.

Update the weights and offsets, respectively (Eq. (19)):

19 $$ \begin{aligned}   W_{{ij}}  &  = W_{{ij}}  + \Delta W_{{ij}}  = W_{{ij}}  + \lambda E_{j} O_{i}  \\    \theta _{j}  &  = \theta _{j}  + \vartriangle \theta _{j}  = \theta _{j}  + \lambda E_{j}  \\  \end{aligned}  $$

where *λ* is the learning rate, with a value of 0–1. When the training reaches a certain number of iterations or the accuracy is higher than a certain value, the training is stopped.

## Passenger satisfaction prediction

**Figure 4:**
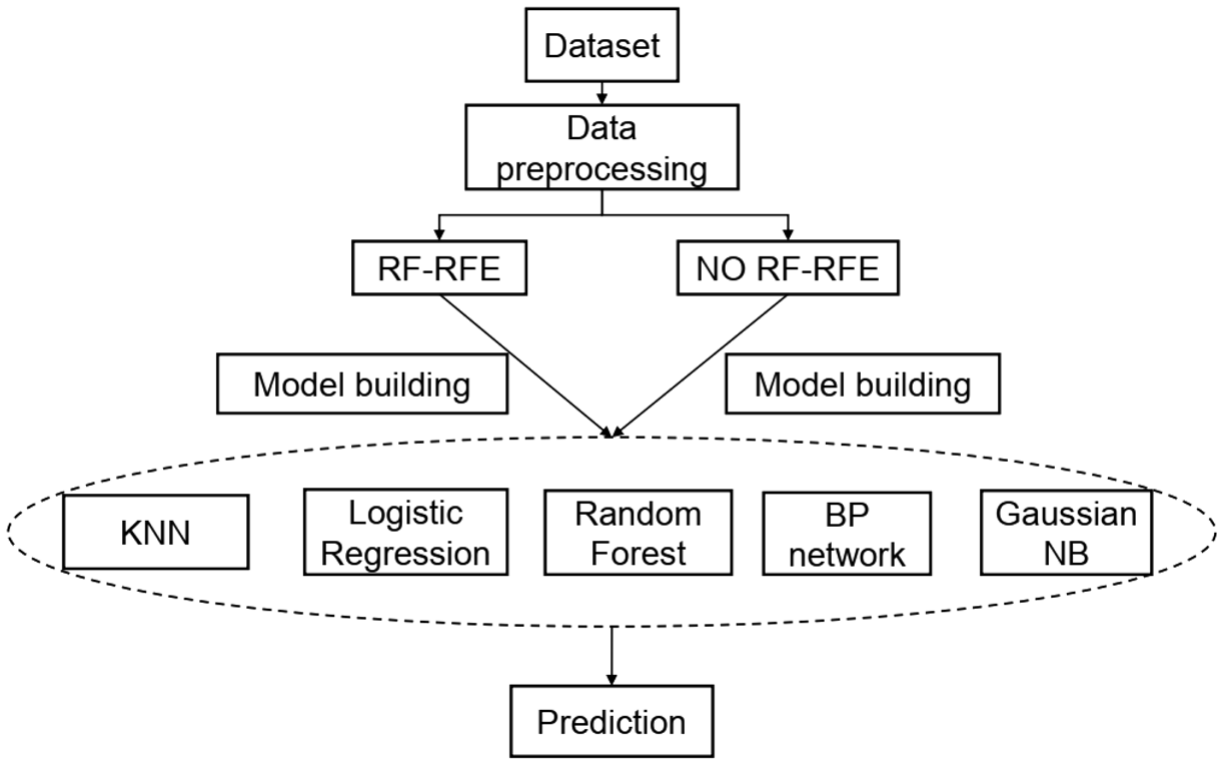
Research framework.

Based on the preliminary selection of features, this study establishes models to predict passenger satisfaction. After comparing the prediction performance of various classification models before and after feature selection, we select the model with the best prediction performance. Figure 4 shows the research framework of the prediction analysis process.

### Data source

The data used in this study are the passenger satisfaction data set of an American airline on Kaggle (https://www.kaggle.com/binaryjoker/airline-passenger-satisfaction). Through the survey of passengers who arrived at the airport in 2015, a sample of 129,880 passengers using the full service of the airline was collected. There are 23 attributes in the data set, of which the input variables include 4 numerical continuous variables, 4 class discrete variables and 14 qualitative sequential variables, indicating the customer's satisfaction with relevant services (0–5 points). The data used in this study mainly contain three dimensions of information, including basic information, flight information and satisfaction information. The constituent elements and the specific variable names and variable attributes are shown in Table 2. The output variables are category variables, that is, passengers' final satisfaction and dissatisfaction or neutral attitude.

**Table 2:** Variable name and attribute.

Variable properties	Variable name	Satisfaction (0–5)	Flight operation quality	Departure arrival time convenient
Numerical type	Age		Ticketing service	Ease of online booking
	Flight distance			online boarding
			Ground service	Gate location
	Departure delay in minutes			Baggage handling
				Checking service
	Arrival delay in minutes		Air service	Inflight Wi-Fi service
				Food and drink
Category type	Gender			Seat comfort
	Type of travel			Inflight entertainment
	Customer type			Onboard service
				Leg room service
	Customer class			Inflight service
				Cleanliness

### Data preprocessing

#### Data cleaning and standardization

There are 393 missing values in the data set. After deleting the missing values, there are 129,487 samples. There are large differences in the value range of the four numerical variables: age, flight distance, departure delay (minutes) and arrival delay (minutes). The data are standardized and transformed into dimensionless values for comparison and weighting between different variables.

#### Correlation test

According to the correlation matrix results, the correlation coefficient between departure delay (departure_delay_in_minutes) and arrival delay (arrival_delay_in_minutes) is as high as 0.964, so the variable of arrival delay is removed.

### Feature selection based on RF-RFE

**Figure 5:**
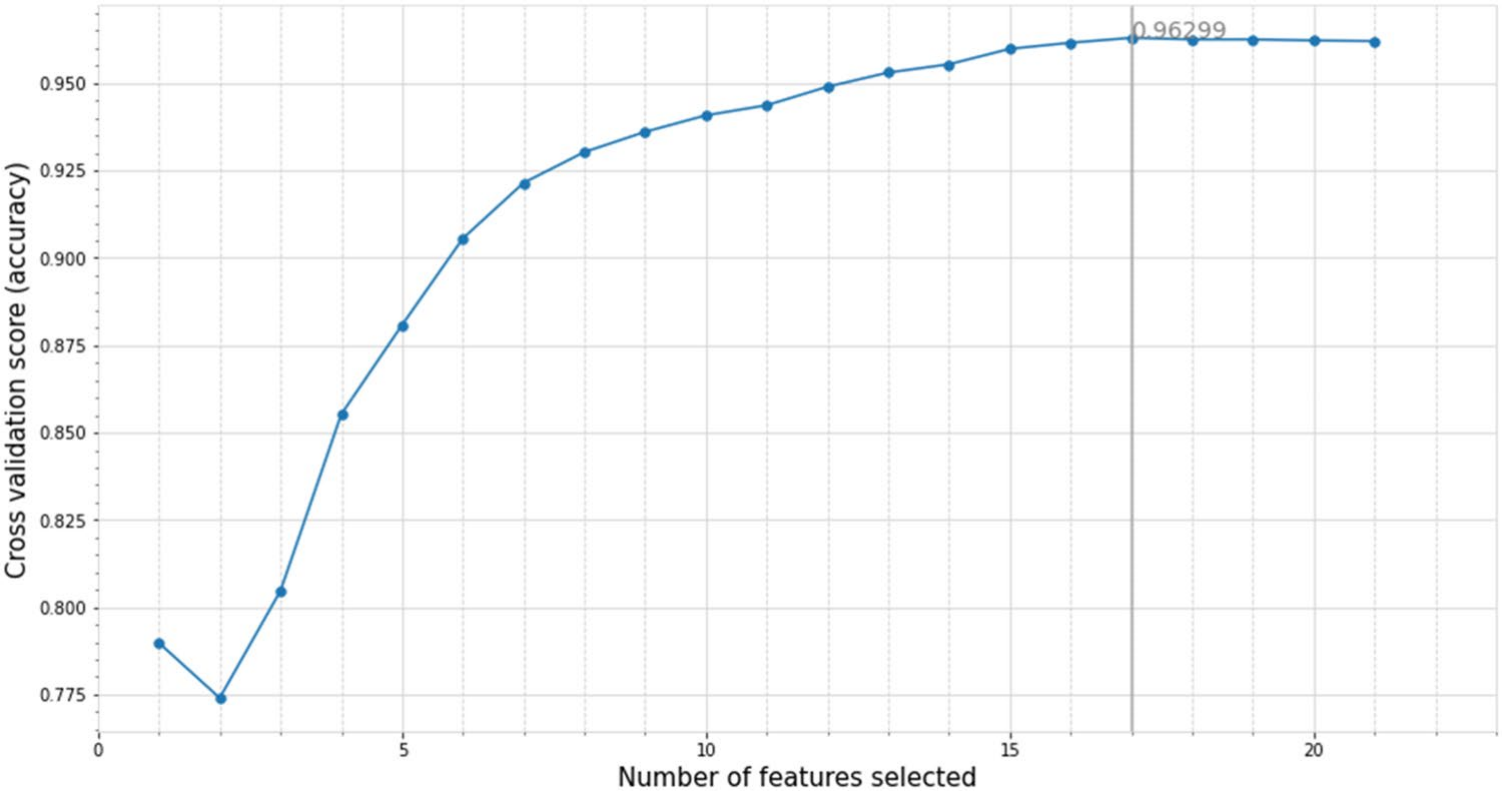
Feature selection results based on RF-RFE.

In this study, the combination of RFE and the cross validation method is used to calculate the selected feature set in each RFE stage for cross validation. Taking the accuracy as the evaluation criterion, the number of features with the highest accuracy and the corresponding feature subset are finally determined. The RF-RFE feature selection results are shown in Fig. 5. The broken line diagram can intuitively judge the accuracy results obtained by the number of different feature subsets, in which the number of features in the feature subset with the highest accuracy is 17, and the classification accuracy of the test set is 0.963.

### Model prediction

#### Model evaluation index

To evaluate the classification performance of the classifier used in the research, five evaluation indexes are introduced: accuracy, precision, recall, F value and AUC value. For the binary classification problem, the sample can be divided into four cases according to the combination of its real category and the category predicted by the classifier: true positives (TP), false positives (FP), true negatives (TN) and false negatives (FN). Obviously, TP + FP + TN + FN = the total number of test sets.

Accuracy is the most commonly used performance measure in classification tasks. It refers to the proportion of the number of correctly classified samples to the total number of samples for a given test set. Its equation is Eq. (20):

20 $$Accuracy=\frac{TP+TN}{TP+TN+FP+FN}$$

Precision refers to the proportion of samples whose real situation is positive in the samples determined as positive by the classifier for a given test set. Its equation is Eq. (21):

21 $$Precision=\frac{TP}{TP+FP}$$

The recall rate refers to the proportion of samples determined as positive by the classifier in all positive samples for a given test set. The equation is Eq. (22):

22 $$Recall=\frac{TP}{TP+FN}$$

In general, the precision and recall contradict each other. The higher the precision is, the lower the recall; when the recall is high, the precision is often low. To comprehensively consider the precision and recall, the F value is introduced to more comprehensively evaluate the classification performance of a classifier. F is the weighted harmonic average based on precision and recall, and the equation is as follows Eq. (23):

23 $$\begin{aligned}\frac{1}{F}&=\frac{1}{1+{\beta }^{2}}\cdot\left(\frac{1}{Precision}+\frac{{\beta }^{2}}{Recall}\right)\\ F&=\frac{\left(1+{\beta }^{2}\right)\times Precision\times Recall}{\left({\beta }^{2}\times Precison\right)+Recall}\end{aligned}$$

where $\upbeta$ reflects the relative importance of precision to recall. When $\upbeta =1$, that is, the precision is as important as the recall, the F value is the commonly used F1 value. Its equation is Eq. (24):

24 $$F=\frac{2\times Precision\times Recall}{Precision+Recall}$$

**Figure 6:**
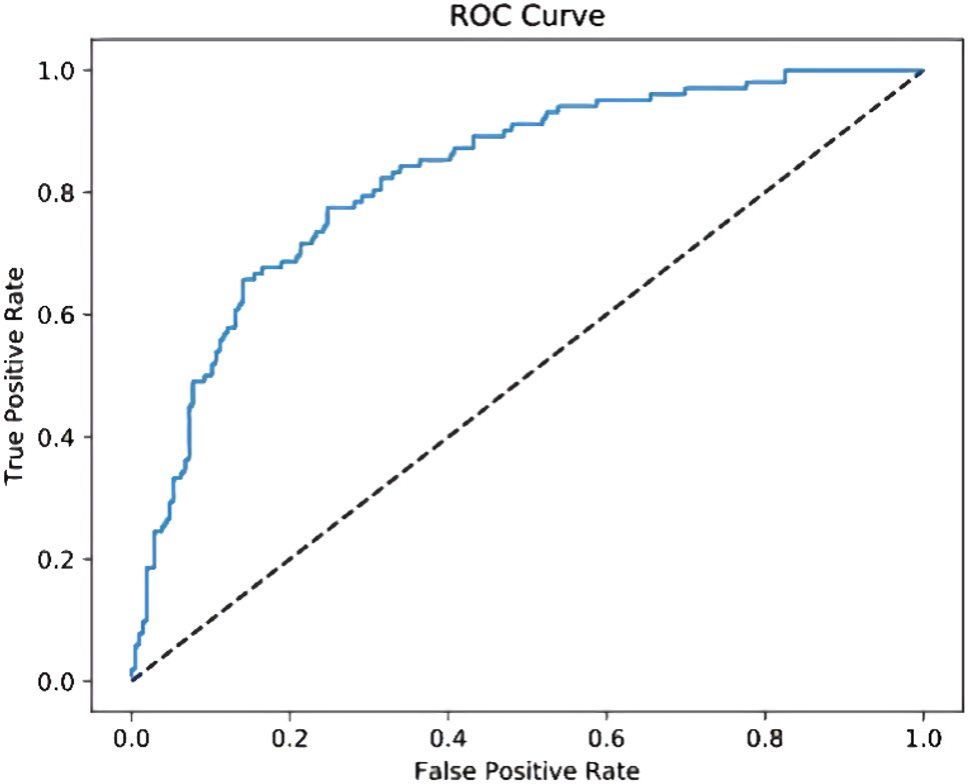
ROC curve.

The ROC curve sorts the samples according to the prediction results of the classifier, forecasts the samples one by one as positive examples in this order, and draws the ROC curve as shown in Fig. 6 with "FP rate" as the abscissa and "TP rate" as the ordinate, and the AUC value is the area under the ROC curve. The AUC value can directly evaluate the quality of the classifier. The larger the AUC value is, the better the classifier performance.

#### Model prediction results

Using the feature subset (including 17 features) after RF-RFE feature selection, a classification model is constructed to predict the satisfaction of passengers in the test set, including KNN, LR, GNB and RF. During BPNN training, the activation function is set as "ReLU", the L2 penalty (regularization term) parameter is 0.0001, and the solver is the default "Adam", which can work well in terms of training time and verification score in the face of relatively large data sets.

This study uses five evaluation indexes, accuracy, precision, recall, F1 value and AUC value, to compare the classification performance of the classifier vertically and horizontally. Table 3 shows the classification performance of each classification model before and after RF-RFE feature selection.

**Table 3:** Model evaluation results.

	Models	Accuracy	Precision	Recall	F1 score	AUC
No RF-RFE	KNN	0.930	0.944	0.890	0.917	0.925
	LR	0.873	0.867	0.835	0.851	0.869
	GNB	0.865	0.861	0.821	0.841	0.860
	RF	0.962	0.972	0.940	0.955	0.960
	BP	0.959	0.964	0.940	0.952	0.957
RF-RFE	KNN	0.934	0.942	0.903	0.922	0.930
	LR	0.872	0.865	0.834	0.849	0.867
	GNB	0.866	0.863	0.819	0.840	0.860
	RF	0.963	0.973	0.942	0.957	0.961
	BP	0.954	0.936	0.960	0.948	0.955

Among all classifiers, the RF model based on RF-RFE feature selection performs best, and the five indexes (accuracy, precision, recall, F1 value and AUC value) are greater than those of the other classifiers. The indexes (except precision) of the KNN model increased after RF-RFE. The five indexes of the logistic model decreased slightly after RF-RFE. After RF-RFE feature selection, the two indexes (accuracy and precision) of the GNB model increase, and the other indexes are slightly lower than those of the model without feature selection. After feature selection, the indexes (except recall) of the BPNN model are slightly reduced. In general, in the overall comparison of the five classification models, RF is better than BPNN, followed by KNN and logistics, and GNB is the worst model.

## Discussion on important variables

In this study, the most important factors affecting passenger satisfaction were selected by the feature selection method. In “Model prediction” section, we used RF-RFE to make a preliminary selection of the feature set and initially selected 17-dimensional features from 22-dimensional features, but the number of features was still large. Therefore, we used the logistic regression model to further select the features in 4.1, and the features with the largest coefficients were further analyzed.

### Extracting variables from logistic model

**Figure 7:**
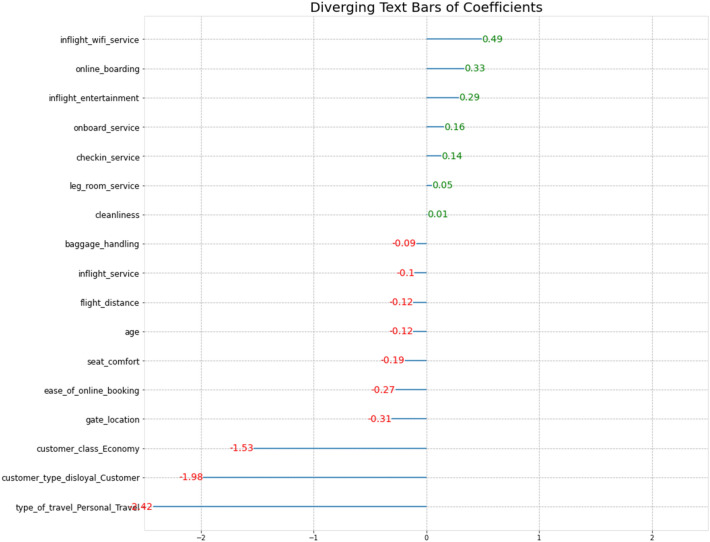
LR coefficient.

The LR model is constructed based on RF-RFE feature selection, and further feature extraction is carried out through LR. Figure 7 shows the coefficient of the LR variable. The results show that except for the cleanliness of the variable, all other variables passed the significance test. In LR, when the features are standardized, the greater the absolute value of the coefficient, the more important the feature is. If the coefficient is positive, the characteristic is positively correlated with the probability that the output is 1; in contrast, it is positively correlated with the probability that the output is 0. Among the 17 variables after RF-RFE feature extraction, the first 5 variables with the largest absolute value of the coefficient are type_ of_ travel_ personal travel, customer_ type_ disloyal customer, customer_ class_ economy, inlight_ Wi-Fi_ service, and online_ boarding.

### Analysis and suggestions

In airline competition, service quality is the key to winning the choice of passengers. Airlines should adopt a customer-oriented service evaluation method to improve service by understanding which service strategies are the most effective strategies to win more passengers. Based on the big data of airline passengers, the research results of this study give the priority of services that airlines need to pay attention to and give suggestions for services with higher priority through the extraction of important variables.

The five important variables extracted from RF-RFE-logistics are travel type (personal travel and business travel), customer type (loyal and not-loyal), customer class (economy class and business class), inflight Wi-Fi service and online boarding. The travel type is uncontrollable, so we further analyze the three aspects of customer type and customer class, in-flight wi-fi, and online boarding.

#### Customer type and customer class

**Figure 8:**
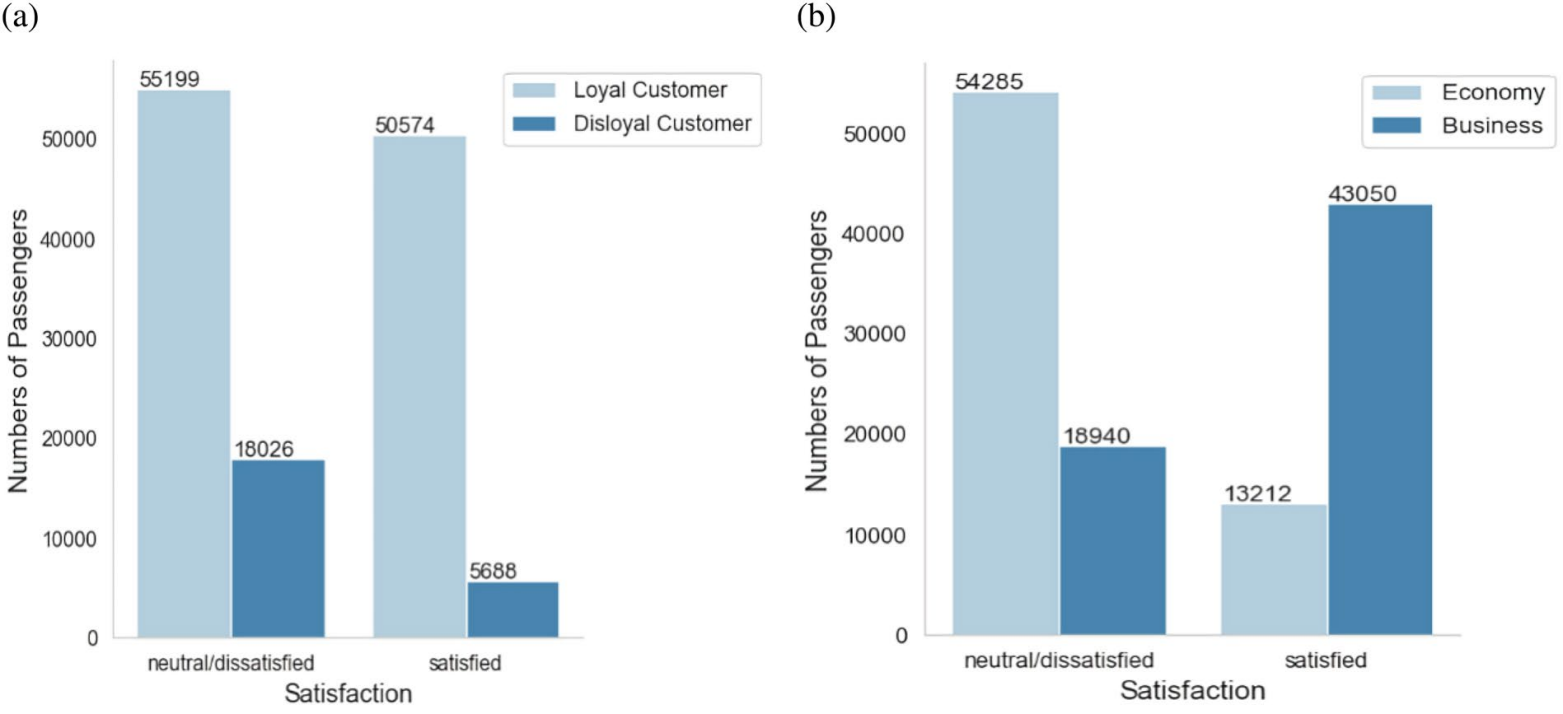
Distribution of people by customer type: (**a**) by passenger type; (**b**) by customer class.

Figure 8 shows the distribution of satisfaction according to different passenger types and different cabin types. As seen from the figure, loyal customers account for more than 80%. Among loyal customers, the number of satisfied passengers is close to that of neutral or dissatisfied passengers. In the 1980s, an airline first proposed the Frequent Flyer Program (FFP). In 1994, Air China first implemented it in China. It is a plan proposed by airlines to reduce the risk of losing business and the loss of an existing customer base. It usually includes selling points and mileage to project partners to offset air tickets, upgrades or other rewards. For more advanced members, there are additional points as an incentive for high-value passengers. Since the cost of developing a new customer is often higher than that of maintaining a loyal old customer, FFP members of airlines are rapidly becoming the core competitiveness of business development. Therefore, airlines should invest more energy to improve frequent flyer plans, improve passenger satisfaction of loyal users and increase user stickiness.

When the satisfaction level is divided according to customer class, it can be found that the overall satisfaction of economy class passengers is significantly lower than that of business class passengers. Most business class passengers are satisfied with the service, while most economy class travelers tend to be neutral or dissatisfied with the service.

**Figure 9:**
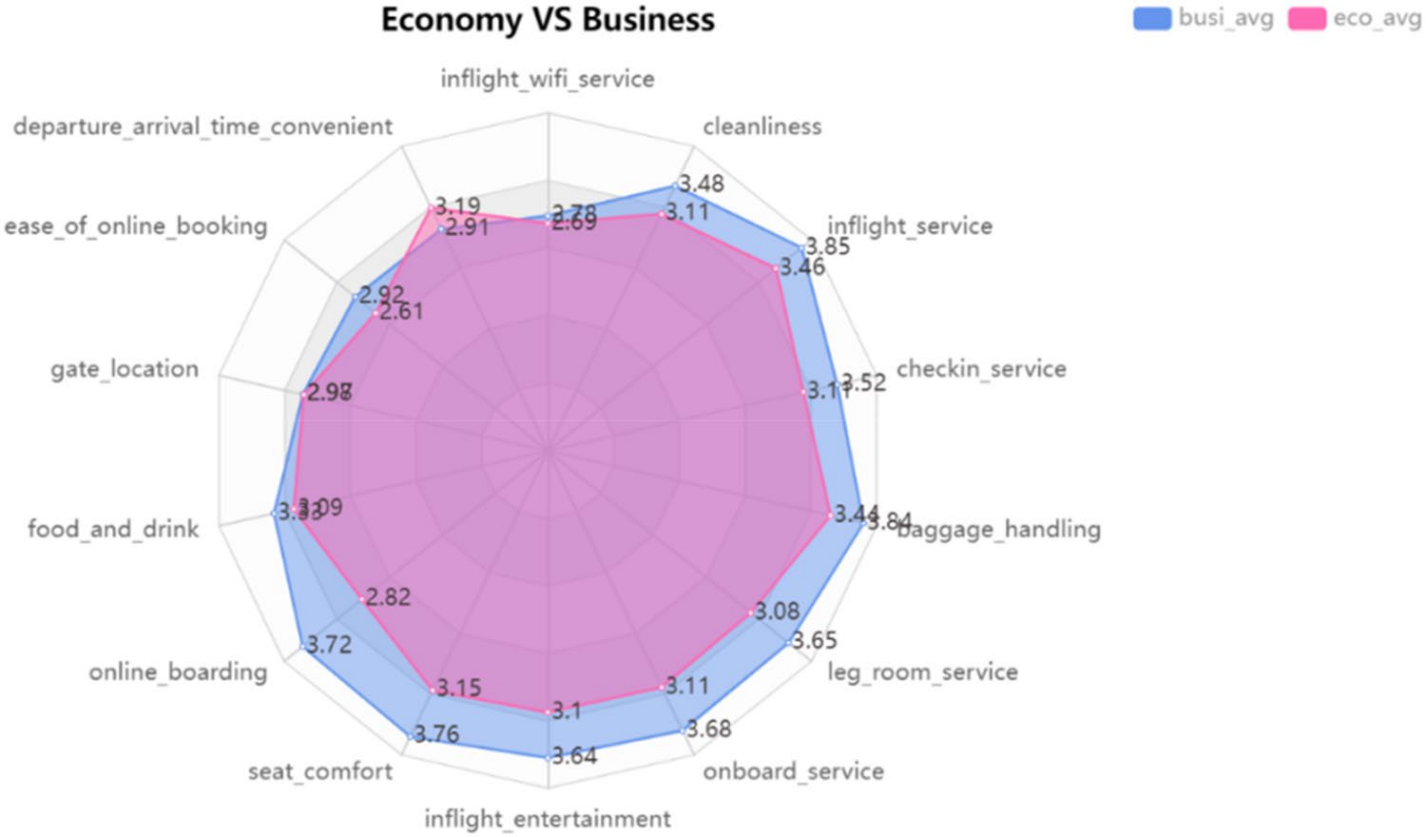
Average score of satisfaction.

According to the customer class, 14 variables related to satisfaction, such as seat comfort and online boarding service, are compared to determine the main factors affecting the difference in passenger satisfaction of different class types. Figure 9 radar chart shows the average satisfaction score of 14 variables for passengers in different classes. The average scores of business class passengers and economy class passengers on seat comfort, leg extension space, on-board entertainment and online boarding are very different, but there is no significant difference in food and drinks.

#### Inflight Wi-Fi service

The result of RF-RFE-LR shows that the most important type of service is inflight Wi-Fi service, and its satisfaction score is the lowest among all services. Mobile Internet has become indispensable in daily life. People's diversified lifestyle is in sharp contrast to traditional monotonous entertainment. Passengers' demand for connecting personal electronic devices to the Internet is becoming increasingly stronger. If airlines can provide Wi-Fi services and provide free internet access or reasonable Wi-Fi prices, they can attract more passengers. According to the survey of INMARSAT in 2016, more than half (54%) of passengers will choose Wi-Fi instead of on-board meals. According to the survey in 2018, onboard Wi-Fi is listed as the fourth most important factor when passengers choose airlines around the world after airline reputation, freely checked baggage and additional leg space. If the flight provides high-quality Wi-Fi, 67% of passengers said they would rebook the airline ticket. In addition, passengers carrying their own personal electronic equipment can also reduce the investment and maintenance of cabin entertainment equipment such as electronic displays and other hardware. The improvement of cabin facilities should be combined with the development of the times. Increasing the popularity of cabin Internet is the general trend. Therefore, from the perspective of improving passenger satisfaction, airlines have good reasons to provide and upgrade onboard Wi-Fi services.

#### Online boarding

Furthermore, the service that needs to be improved is online boarding. CAAC proposed the concept of "paperless travel" at the 2018 national civil aviation work conference, replacing paper boarding passes with paperless forms such as electronic boarding passes. With ID cards and e-tickets, you can check in directly on the Internet platform without printing boarding passes. It can not only realize the paperless boarding system but also save passengers waiting in line at the counter for check-in, save passengers' time and energy, and make the boarding process faster and more convenient. Therefore, in addition to providing ticket booking, seat reservation, change reservation and meal ordering services, the airline travel service app can add the service of check-in and printing electronic boarding passes in the design, optimize the user boarding experience, improve the competitiveness of the airline travel service app and increase user stickiness. Currently, due to the development of artificial intelligence, the "one card mode" combined with face recognition is possible. Passengers only need to use the second-generation ID card and face recognition camera to realize the unification of ticket information to make each process of passengers at the airport more efficient and improve their travel experience.

## Conclusion

We proposed a RF-RFE-Logistic feature selection model to extract the influencing factors of passenger satisfaction based on airline passenger satisfaction survey information. We first use the RF-RFE algorithm to preliminarily extract a feature subset containing 17 variables and use a variety of classification learning algorithms to predict passenger satisfaction. RF on this feature subset shows the best classification performance (accuracy: 0.963, precision: 0.973, recall: 0.942, F1 value: 0.957, AUC value: 0.961). Then, we use a logistic model trained on the feature subset selected by RF-RFE to further extract the important variables affecting passenger satisfaction. Finally, the satisfaction of different passenger types and class types are compared and analyzed, and suggestions are given from the perspectives of online boarding and onboard Wi-Fi services.

There are also some deficiencies in this study. The evaluation indicators of passenger satisfaction surveys in the data set used in this study are not sufficient. In addition, we used the default parameters in the prediction model and did not consider the prediction results of different parameters. Based on the above limitations, we suggest that: (1) the ground service can also include aspects such as baggage claim speed, transfer service, etc. In addition, satisfaction level of related services under abnormal flight conditions can also be added; (2) pay attention to the optimized model parameters; (3) several other variables also affect passenger satisfaction to a certain extent, which should not be completely ignored.

## Data Availability

Data and methods used in the research have been presented in sufficient detail in the paper.
